# Physical exercise reverses immuno-cold tumor microenvironment via inhibiting SQLE in non-small cell lung cancer

**DOI:** 10.1186/s40779-023-00474-8

**Published:** 2023-08-18

**Authors:** Zhi-Wen Luo, Ya-Ying Sun, Wei Xia, Jun-Ying Xu, Dong-Jing Xie, Chun-Meng Jiao, Ji-Ze Dong, Hui Chen, Ren-Wen Wan, Shi-Yi Chen, Jie Mei, Wen-Jun Mao

**Affiliations:** 1 Department of Sports Medicine, Huashan Hospital Affiliated to Fudan University, Shanghai, 200040 China; 2 Department of Sports Medicine, Shanghai General Hospital, Shanghai, 200080 China; 3 Department of Critical Care Medicine, the Affiliated Wuxi People’s Hospital of Nanjing Medical University, Wuxi, 214023 Jiangsu China; 4 Department of Oncology, the Affiliated Wuxi People’s Hospital of Nanjing Medical University, Wuxi, 214023 Jiangsu China; 5 Guangdong Pharmaceutical University, Guangzhou, 510006 China; 6 Institute of Acupuncture Research, Institutes of Integrative Medicine, Fudan University, Shanghai, 200433 China; 7 Department of Cardiothoracic Surgery, the Affiliated Wuxi People’s Hospital of Nanjing Medical University, Wuxi, 214023 Jiangsu China

**Keywords:** Physical exercise, Non-small cell lung cancer (NSCLC), Squalene epoxidase (SQLE), Tumor immune microenvironment (TIME)

Dear Editor,

Physical exercise has been shown to be associated with reduced cancer incidence and cancer-associated mortality [1, 2], but the underlying mechanisms are obscure. Immunometabolic regulation has emerged as one of the most prominent mechanisms explaining the effects of exercise on cancer [1, 2]. Physical exercise primarily lowers blood cholesterol and triglycerides, and protects against cardiovascular diseases [3]. However, whether physical exercise can modulate cholesterol metabolism in tumor cells is currently unknown.

Metabolic reprogramming is one of the hallmarks of cancer, and metabolic dysregulation critically contributes toward oncogenesis and tumor progression [4]. Being a common phenomenon associated with both physical exercise and cancer, metabolic regulation is one of the critical mechanisms that mediates the anticancer effects of physical exercise.

Cholesterol, the major sterol in mammalian cell membranes, maintains cell integrity and intracellular homeostasis. Previously, we found that certain cholesterol-related genes were more active in non-small cell lung cancer (NSCLC), which is an immuno-cold tumor. Blocking cholesterol production by treating these cancer cells with 3-hydroxy-3-methylglutaryl-coenzyme A reductase (HMGCR) inhibitors induced an elevated immune response to the inflamed tumor immune microenvironment (TIME) [5]. However, the complexity of cholesterol biosynthesis warrants the discovery of more interventions.

In this study, we first explored the effects of physical exercise on gene expression in tumors. Re-analyzing the GSE62628 dataset, we found that physical exercise significantly modulated the transcriptome of mouse melanoma cells (Fig. 1a, Additional file 1: Fig. S1). The upregulated genes were associated with immune-related processes (Additional file 1: Fig. S2a, b), and the downregulated genes were associated with cholesterol biosynthesis (Additional file 1: Fig. S2c, d). We further validated this result using a mouse lung cancer model (Fig. 1b), and found that exercise notably inhibited tumor growth (Fig. 1c, Additional file 1: Fig. S3). Next, we used mass cytometry to analyze changes in the TIME (Additional file 1: Fig. S4). Exercise triggered the infiltration of antitumor immune cells, such as CD8^+^ T cells, M1 macrophages, and B cells, while inhibiting the infiltration of pro-tumor immune cells, such as myeloid-derived suppressor cells (Fig. 1d, Additional file 1: Fig. S5). T cell subset analysis revealed that the proportions of naïve and activated T cells were significantly increased, further evidencing that exercise modulated the TIME (Additional file 1: Fig. S6). The increased proportions of CD8^+^ T cells and M1 macrophages were verified using immunofluorescence analysis of mouse tumor tissues (Fig. 1e). We also validated the effect of exercise on cholesterol metabolic reprogramming in mouse tumor tissues. Exercise significantly inhibited squalene epoxidase (SQLE) expression, but did not affect HMGCR expression (Fig. 1e). Overall, we found that physical exercise reversed the immuno-cold TIME and inhibited cholesterol metabolism.

**Fig. 1:**
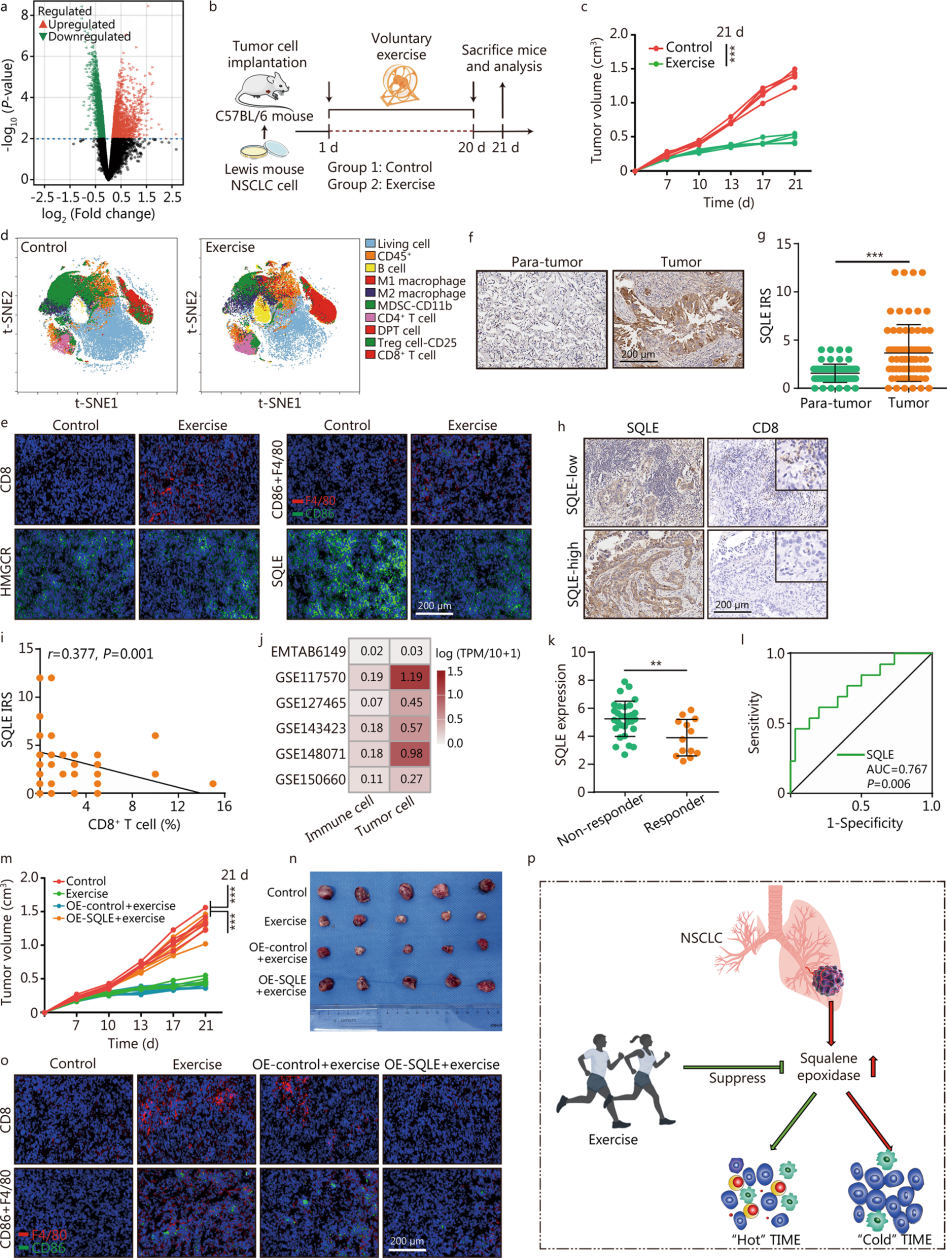
Physical exercise “heats” immuno-cold tumors by inhibiting cholesterol synthesis. **a** Overview of DEGs between tumors in mice with or without voluntary wheel running. **b** Schematic protocol of the experimental procedures in C57BL/6 mice. **c** Tumor growth in Lewis carcinoma-bearing mice with or without exercise (*n* = 5 per group). **d** t-SNE plots of 50,000 cells per group colored using PhenoGraphcluster. **e** Immunofluorescence revealing the infiltration of CD8^+^ T cells and M1 macrophages, and the expression of HMGCR and SQLE. Representative images demonstrating SQLE expression in para-tumor and tumor tissues using anti-SQLE staining (**f**) and semi-quantitative analysis (**g**). **h** Representative images showing CD8 expression in the high- and low-SQLE groups. **i** Semi-quantitative analysis of the correlation between SQLE and CD8 expression. **j** Expression levels of SQLE in different cell types from NSCLC tissues in six scRNA-seq datasets. **k** and **l** Expression of SQLE in patients with different immunotherapy responses and ROC analysis of the predictive value of SQLE (*n* = 30 in the non-responder group, *n* = 13 in the responder group). Data obtained by merging the GSE126044 and GSE135222 datasets. **m** Tumor growth curve and SQLE overexpression in tumors from mice with exercise (*n* = 5 per group). **n** Representative images showing the tumors harvested from Lewis carcinoma-bearing mice (*n* = 5 per group). **o** Representative images showing the infiltration of CD8^+^ T cells and M1 macrophages in the above tumors. **p** Schematic diagram of this study: exercise heats up the TIME by suppressing SQLE expression. ^**^P < 0.01, ^***^P < 0.001. MDSC myeloid-derived suppressor cell, IRS immunoreactivity score, DEGs differentially expressed genes, HMGCR 3-hydroxy-3-methylglutaryl-coenzyme A reductase, SQLE squalene epoxidase, NSCLC non-small cell lung cancer, scRNA-seq single-cell RNA sequencing, OE overexpression, TIME tumor immune microenvironment, ROC receiver operating characteristic, t-SNE t-distributed Stochastic Neighbor Embedding, DPT double positive T cells

To identify the critical gene controlling cholesterol metabolism in NSCLC, we investigated the expression and prognostic value of cholesterol biosynthesis-related genes in NSCLC using The Cancer Genome Atlas (TCGA) dataset. Most genes were dysregulated in NSCLC (Additional file 1: Fig. S7a), but only the upregulation of SQLE was associated with poor prognosis (Additional file 1: Fig. S7b). Given its critical role in catalyzing the primary oxygenation step of sterol biosynthesis and its prognostic significance, we identified SQLE as a potential rate-limiting enzyme in cholesterol production that warranted further analysis. Immunohistochemistry (IHC) analysis of human NSCLC tissues was performed to further validate SQLE expression in NSCLC, and the result confirmed that SQLE was notably upregulated (Fig. 1f, g). These findings revealed that SQLE was a potential oncogene in NSCLC.

Next, we assessed the immuno-correlation of SQLE in NSCLC using the TCGA dataset. High SQLE expression was related to the downregulation of most immunomodulators (Additional file 1: Fig. S8a, b). In addition, SQLE expression was positively correlated with tumor purity and negatively correlated with immune cell infiltration (Additional file 1: Fig. S8c, d). We also validated the negative correlation between SQLE expression and CD8^+^ T cell abundance using an in-house NSCLC cohort (Fig. 1h, i). However, we only observed the correlations between SQLE expression and the immuno-cold TIME; the effects of SQLE on TIME functionality are still unknown. Furthermore, the Single Cell Expression Atlas of human NSCLC tumors uncovered that SQLE was enriched in tumor cells (Fig. 1j, Additional file 1: Fig. S9). Given its negative immuno-correlation in NSCLC, we speculated that SQLE may be associated with immunotherapeutic responses. In a combined public cohort, we found that SQLE was downregulated in cases with a poor response, and this was also validated in our recruited NSCLC cohort receiving immune checkpoint inhibitors therapy (Fig. 1k, l, Additional file 1: Fig. S10). Moreover, SQLE overexpression notably reversed exercise-mediated tumor inhibition in vivo (Fig. 1m-o, Additional file 1: Fig. S11). Overall, SQLE expression was related to the immuno-cold TIME and reversed physical exercise-induced tumor inhibition and TIME activation.

In summary, physical exercise inhibited tumor progression by significantly downregulating SQLE, which modulated the inflamed TIME and enhanced immune checkpoint inhibitors therapy. In addition, SQLE expression was related to poor prognosis and the immuno-cold TIME (Fig. 1p). Overall, we have clarified the important role of SQLE in maintaining the immuno-cold phenotype in NSCLC, and propose physical exercise as an intervention for SQLE. However, these observations were made in mice; prospective clinical trials in humans are warranted before we decide to sensitize immunotherapy using exercise therapy.

### Supplementary Information

**Additional file 1**. Materials and methods. **Fig. S1** A total of 1980 genes were downregulated and 1445 genes were upregulated. **Fig. S2** Reactome analysis of DEGs in the GSE62628 dataset. **Fig. S3** Weight of the harvested tumors in tumor-bearing mice with or without exercise (*n* = 5 per group). **Fig. S4** t-SNE plots of normalized marker expression for classifying tumor cells from mice in the control and exercise groups. **Fig. S5** Marker expression and cell proportion of different cell subpopulations. **Fig. S6** Changes in the T cell subset proportions in mice tumors from the control and exercise groups. **Fig. S7** Expression and prognostic value of genes related to cholesterol synthesis in NSCLC tissues. **Fig. S8** Correlation between SQLE expression and TIME features. **Fig. S9** Expression levels of SQLE in different cell types in NSCLC tissues in the GSE117570 dataset.** Fig. S10** Representative images depicting SQLE expression in patients with different immunotherapy responses and semi-quantitative analysis of the predictive value of SQLE (*n* = 14 in the non-responder group, *n* = 8 in the responder group). **Fig. S11** Weight of the harvested tumors from tumor-bearing mice (*n* = 5 per group). **Table S1** A list of the antibodies and reagents used in this research

## Data Availability

All data supporting the results of this study are shown in this published article and supplementary documents. In addition, original omics data for bioinformatics analysis could be obtained from corresponding platforms.

## Abbreviations

HMGCR: 3-Hydroxy-3-methylglutaryl-coenzyme A reductase
IHC: Immunohistochemistry
NSCLC: Non-small cell lung cancer
TIME: Tumor immune microenvironment
SQLE: Squalene epoxidase
TCGA: The Cancer Genome Atlas
