# Supplementary material for: Physical exercise reverses immuno-cold tumor microenvironment via inhibiting SQLE in non-small cell lung cancer
Source: Mil Med Res. 2023 Aug 18;10:39. doi: 10.1186/s40779-023-00474-8 (PMC10436398; doi:10.1186/s40779-023-00474-8)
Supplement: Supplementary file 1 — Additional file 1. Materials and methods. Fig. S1 A total of 1980 genes were downregulated and 1445 genes were upregulated. Fig. S2 Reactome analysis of DEGs in the GSE62628 dataset. Fig. S3 Weight of the harvested tumors in tumor-bearing mice with or without exercise (n = 5 per group). Fig. S4 t-SNE plots of normalized marker expression for classifying tumor cells from mice in the control and exercise groups. Fig. S5 Marker expression and cell proportion of different cell subpopulations. Fig. S6 Changes in the T cell subset proportions in mice tumors from the control and exercise groups. Fig. S7 Expression and prognostic value of genes related to cholesterol synthesis in NSCLC tissues. Fig. S8 Correlation between SQLE expression and TIME features. Fig. S9 Expression levels of SQLE in different cell types in NSCLC tissues in the GSE117570 dataset. Fig. S10 Representative images depicting SQLE expression in patients with different immunotherapy responses and semi-quantitative analysis of the predictive value of SQLE (n = 14 in the non-responder group, n = 8 in the responder group). Fig. S11 Weight of the harvested tumors from tumor-bearing mice (n = 5 per group). Table S1 A list of the antibodies and reagents used in this research [file 40779_2023_474_MOESM1_ESM.pdf]

Materials and methods

Public datasets acquisition

The standardized datasets of lung adenocarcinoma and lung squamous cell carcinoma from TCGA were downloaded from the website of the University of California, Santa Cruz (http://www.xenabrowser.net/) [TCGA Lung Cancer (LUNG) cohort]. In addition, the GSE126044 and GSE135222 datasets, which comprised RNA-sequencing data from NSCLC patients receiving immunotherapy, were obtained from Gene Expression Omnibus (http://www.ncbi.nlm.nih.gov/geo/). For incorporating NSCLC samples in the GSE126044 and the GSE135222 cohorts, we first merged the two datasets using the "sva" package in R [1], and removed the batch effects using empirical Bayes methods [2].

Analysis of tumor immune microenvironment features

The features of the TIME were mainly defined by the expression levels of chemokines and receptors, the levels of tumor-infiltrating lymphocytes, and tumor purity [3,4]. Immunomodulators included chemokines and receptors, tumor-infiltrating lymphocytes were calculated using the Tumor Immune Estimation Resource algorithm [5], and purity data were obtained from previous research [6]. In addition, the expression of SQLE in various cell types in NSCLC was explored using the Tumor Immune Single-cell Hub tool (http://tisch.comp-genomics.org/home/).

Reagents and antibodies

The sources of all reagents and antibodies used in this research are described in Additional file 1: Table S1.

Clinical cohorts

NSCLC tissue microarrays (TMAs) were purchased from Outdo BioTech (Shanghai, China; Cat. HLugA150CS01). The HLugA150CS01 cohort contained 75 NSCLC samples and paired para-tumor samples. Detailed clinicopathological characteristics were obtained from Outdo BioTech. Ethical approval for using the TMAs was granted by the Clinical Research Ethics Committee at Outdo Biotech (YBM-05-02). In addition, 22 NSCLC patients receiving anti-programmed cell death 1/anti-programmed cell death ligand 1 monotherapy or a combination of chemotherapies were recruited by the Affiliated Wuxi People's Hospital of Nanjing Medical University from January 2019

to February 2022. The therapeutic responses, evaluated according to the RECIST 1.1 criterion, were demarcated into four categories: complete response, partial response, stable disease, and progressive disease [7]. Ethical approval for the collection of samples was granted by the Clinical Research Ethics Committee at the Affiliated Wuxi People's Hospital of Nanjing Medical University (KY21126).

IHC and semi-quantitative evaluation

IHC staining was performed on the above TMAs and tissue slides. The primary antibodies used in this study were anti-SQLE (1:400, Cat. 12544-1-AP, ProteinTech, China) and anti-CD8 (1:200, Cat. GT2280, GeneTech, China). Antibody staining was visualized using diaminobenzidine and hematoxylin counterstain, and captured using Aperio Digital Pathology Slide Scanners [8]. The stained sections were independently evaluated by two pathologists using previously described criteria [9]. Briefly, the percentage of positively stained cells was scored from 0 – 4: 0 (< 5%), 1 (6 – 25%), 2 (26 – 50%), 3 (51 – 75%), and 4 (> 75%). The staining intensity was scored from 0 – 3: 0 (negative), 1 (weak), 2 (moderate), and 3 (strong). The immunoreactivity score was obtained by multiplying the percentage of positive cells with the staining intensity. The quality of this score was ensured by randomly evaluating these sections and blinding the two pathologists to the numbering and assignment of the sections.

Cell culture and lentiviral transfection

The mouse Lewis lung carcinoma cell line was purchased from KeyGEN (Nanjing, China) and cultured at 37 °C with 5% CO_2 in Dulbecco's Modified Eagle Medium with 10% fetal bovine serum. SQLE was overexpressed using lentiviral transfection according to the manufacturer's instructions [10]. Cells were seeded at 30% confluence in 6-well plates. After 24 h, cells in each well were transfected with virus solution (1×10^7 transduction units/ml) for 16 – 24 h, incubated at 37 °C for 72 h, and assessed under a fluorescence microscope. Finally, 2 μg/ml puromycin was added to the complete medium to select stably transfected cell lines.

In vivo analysis

To investigate the effects of physical exercise on tumors, we first downloaded the GSE62628 dataset from Gene Expression Omnibus (http://www.ncbi.nlm.nih.gov/geo/). It included gene expression profiles of melanoma tumor tissues from exercise and control groups of mice. The "limma" package in R was used to screen differentially expressed genes, and those with a false discovery rate < 0.05

were subjected to gene set enrichment analysis using the Database for Annotation, Visualization, and Integrated Discovery. The top 10 terms were displayed. The c2.cp.reactome.v7.4.symbols.gmt subclass from the Molecular Signatures Database [11] was used as the background.

The in vivo analysis was performed using 4 – 6-week-old C57BL/6 mice (n = 30), which were purchased from Shanghai Laboratory Animal Co., Ltd. and commonly used for NSCLC immunotherapy research [12-14]. The mice were housed and maintained in laminar flow cabinets under specific pathogen-free conditions. All experiments were approved by the Laboratory Animal Ethics Committee at Nanjing Medical University (2022370). Lung cancer xenografts were established by subcutaneously injecting transfected homologous Lewis cells (1×10^7) into the flanks of mice. The injected mice were randomly assigned to either two groups (n = 5; Fig. 1c) or four groups (n = 5; Fig. 1m). Exercise intervention for mice included voluntary running on a wheel [15]. The tumors were regularly measured with calipers every 2 – 3 d. On day 21, the mice were anesthetized with 0.5% sodium pentobarbital solution to remove the tumors [16], which were then photographed and weighed.

After euthanizing the mice, the tumors were carefully dissected and frozen in optimal cutting temperature compound, precooled using liquid nitrogen, and stored at -80°C until they were sectioned. Subsequently, 8-mm-thick serial cryosections were prepared for routine immunofluorescence staining of the target proteins [17,18]. The primary antibodies used were anti-CD8 (1:500, Cat. ab217344, Abcam, UK), anti-CD86 (1:100, Cat. ab220188, Abcam, UK), anti-F4/80 (1:200, Cat. ab6640, Abcam, UK), anti-SQLE (1:500, Cat. 12544-1-AP, ProteinTech, China), and anti-HMGCR (1:500, Cat. 13533-1-AP, ProteinTech, China). Nuclei were visualized using 4’,6-diamidino-2-phenylindole dihydrochloride. Non-direct antibodies and their corresponding secondary antibodies were used, and 5% bovine serum albumin (Cat. GC305006, Servicebio, China) served as the blocking solution. The secondary antibodies used were goat anti-rabbit IgG H&L (Alexa Fluor 488, Cat. ab150077, Abcam, UK) and goat anti-rat IgG H&L (Alexa Fluor 594, Cat. ab150160, Abcam, UK). Images were captured using a fluorescence microscope (ECHO Revolve, USA).

Mass cytometry

When mass spectrometry flow samples were selected, they were randomly numbered and the researcher conducting the on-board testing was unaware about their grouping. Mass cytometry was

performed to analyze the samples obtained from three tumors per group, according to the procedure reported by Burnett et al. [19]. First, the cells were barcoded with distinct combinations of isotopically purified palladium ions that were chelated using isothiocyanobenzyl-EDTA. A total of 1×10^6 cells were treated with stable palladium isotopes in Maxpar Barcode Perm Buffer for 15 min on a shaker, washed twice using phosphate-buffered saline that contained 0.5% bovine serum albumin and 0.02% NaN_3, and pooled into a single 15-ml tube.

The barcoded cells were treated with Fc Receptor Blocking Solution, stained at room temperature for 30 min with a surface antibody cocktail to highlight the target cells, washed twice with the same cell-staining media to remove any excess antibodies, permeabilized with methanol at 4 °C for 10 min, washed twice with the cell-staining media to remove methanol, incubated at room temperature for 1 h with the intracellular cocktail, washed twice with cell-staining media to remove any excess antibodies, treated overnight with 191/193Ir iridium intercalator solution diluted in phosphate-buffered saline with 4% Paraformaldehyde Fix Solution, and washed once with cell-staining media and twice with Cell Acquisition Solution before the mass cytometry run.

The samples were then diluted to approximately 1×10^6 cells/ml in Cell Acquisition Solution containing bead standards and analyzed using a Helios mass cytometer equilibrated with the same. The cytometer collected approximately 0.5×10^6 cell events per sample at an even rate of 400 – 500 events/s, with approximately 1.5×10^6 cell events in each group used for data analysis. The data were analyzed using Cytobank v8.1 (Beckman Coulter), and high-dimensional data were visualized using the t-distributed Stochastic Neighbor Embedding algorithm (5000 iterations, perplexity = 100) [20]. Table S1 provides a list of the antibodies and reagents used in this study.

Statistical analysis

Statistical analysis and figure exhibition were performed using R version 4.0.2 and Graphpad Prism 6.0. Continuous variables were analyzed using the Wilcoxon rank-sum test or the Mann-Whitney test, whereas categorical variables were assessed using the chi-squared test. Pearson's correlation was used to evaluate the correlation between two variables. The prognostic values of categorical variables were assessed using the log-rank test. For all analyses, a P-value < 0.05 was deemed to be statistically significant.

References

1. Leek JT, Johnson WE, Parker HS, Jaffe AE, Storey JD. The sva package for removing batch effects and other unwanted variation in high-throughput experiments. Bioinformatics. 2012;28(6):882-3.
2. Johnson WE, Li C, Rabinovic A. Adjusting batch effects in microarray expression data using empirical Bayes methods. Biostatistics. 2007;8(1):118-27.
3. Mei J, Jiang G, Chen Y, Xu Y, Wan Y, Chen R, et al. HLA class II molecule HLA-DRA identifies immuno-hot tumors and predicts the therapeutic response to anti-PD-1 immunotherapy in NSCLC. BMC Cancer. 2022;22(1):738.
4. Mei J, Cai Y, Xu R, Zhu Y, Zhao X, Zhang Y, et al. Protocol to identify novel immunotherapy biomarkers based on transcriptomic data in human cancers. STAR Protoc. 2023;4(2):102258.
5. Li T, Fu J, Zeng Z, Cohen D, Li J, Chen Q, et al. TIMER2.0 for analysis of tumor-infiltrating immune cells. Nucleic Acids Res. 2020;48(W1):W509-W514.
6. Thorsson V, Gibbs DL, Brown SD, Wolf D, Bortone DS, Ou Yang TH, et al. The immune landscape of cancer. Immunity. 2018;48(4):812-30.e14.
7. Schwartz LH, Litiere S, De Vries E, Ford R, Gwyther S, Mandrekar S, et al. RECIST 1.1-update and clarification: from the RECIST committee. Eur J Cancer. 2016;62:132-7.
8. Mei J, Cai Y, Wang H, Xu R, Zhou J, Lu J, et al. Formin protein DIAPH1 positively regulates PD-L1 expression and predicts the therapeutic response to anti-PD-1/PD-L1 immunotherapy. Clin Immunol. 2023;246:109204.
9. Mao W, Cai Y, Chen D, Jiang G, Xu Y, Chen R, et al. Statin shapes inflamed tumor microenvironment and enhances immune checkpoint blockade in non-small cell lung cancer. JCI Insight. 2022;7(18):e161940.
10. Zhang C, Zhang W, Lu Y, Yan X, Yan X, Zhu X, et al. NudC regulates actin dynamics and ciliogenesis by stabilizing cofilin 1. Cell Res. 2016;26(2):239-53.
11. Liberzon A, Subramanian A, Pinchback R, Thorvaldsdottir H, Tamayo P, Mesirov JP. Molecular signatures database (MSigDB) 3.0. Bioinformatics. 2011;27(12):1739-40.
12. Zhuo Y, Li S, Hu W, Zhang Y, Shi Y, Zhang F, et al. Targeting SNORA38B attenuates tumorigenesis and sensitizes immune checkpoint blockade in non-small cell lung cancer by remodeling the tumor microenvironment via regulation of GAB2/AKT/mTOR signaling pathway. J Immunother Cancer. 2022;10(5):e004113.
13. Zhang X, Wang Y, Fan J, Chen W, Luan J, Mei X, et al. Blocking CD47 efficiently potentiated

therapeutic effects of anti-angiogenic therapy in non-small cell lung cancer. J Immunother Cancer. 2019;7(1):346.

14. Kim DH, Kim H, Choi YJ, Kim SY, Lee JE, Sung KJ, et al. Exosomal PD-L1 promotes tumor growth through immune escape in non-small cell lung cancer. Exp Mol Med. 2019;51(8):1-13.

15. Li HQ, Spitzer NC. Exercise enhances motor skill learning by neurotransmitter switching in the adult midbrain. Nat Commun. 2020;11(1):2195.

16. Ko T, Nomura S, Yamada S, Fujita K, Fujita T, Satoh M, et al. Cardiac fibroblasts regulate the development of heart failure via Htra3-TGF-beta-IGFBP7 axis. Nat Commun. 2022;13(1):3275.

17. Luo Z, Qi B, Sun Y, Chen Y, Lin J, Qin H, et al. Engineering bioactive M2 macrophage-polarized, anti-inflammatory, miRNA-based liposomes for functional muscle repair: from exosomal mechanisms to biomaterials. Small. 2022;18(34):e2201957.

18. Sun Y, Luo Z, Chen Y, Lin J, Zhang Y, Qi B, et al. si-Tgfbr1-loading liposomes inhibit shoulder capsule fibrosis via mimicking the protective function of exosomes from patients with adhesive capsulitis. Biomater Res. 2022;26(1):39.

19. Burnett CE, Okholm TLH, Tenvooren I, Marquez DM, Tamaki S, Munoz Sandoval P, et al. Mass cytometry reveals a conserved immune trajectory of recovery in hospitalized COVID-19 patients. Immunity. 2022;55(7):1284-98.e3.

20. Belkina AC, Ciccolella CO, Anno R, Halpert R, Spidlen J, Snyder-Cappione JE. Automated optimized parameters for t-distributed stochastic neighbor embedding improve visualization and analysis of large datasets. Nat Commun. 2019;10(1):5415.

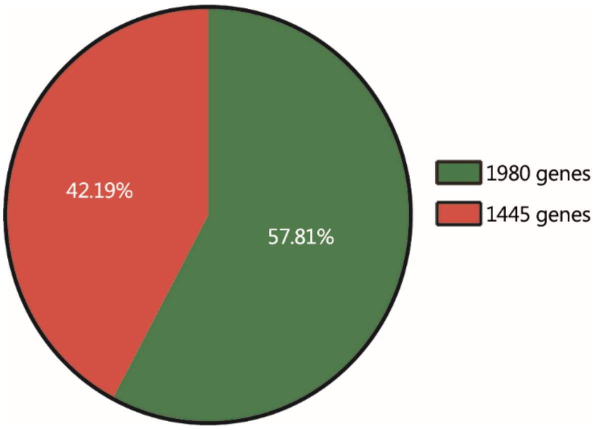

Fig. S1 A total of 1980 genes were downregulated and 1445 genes were upregulated

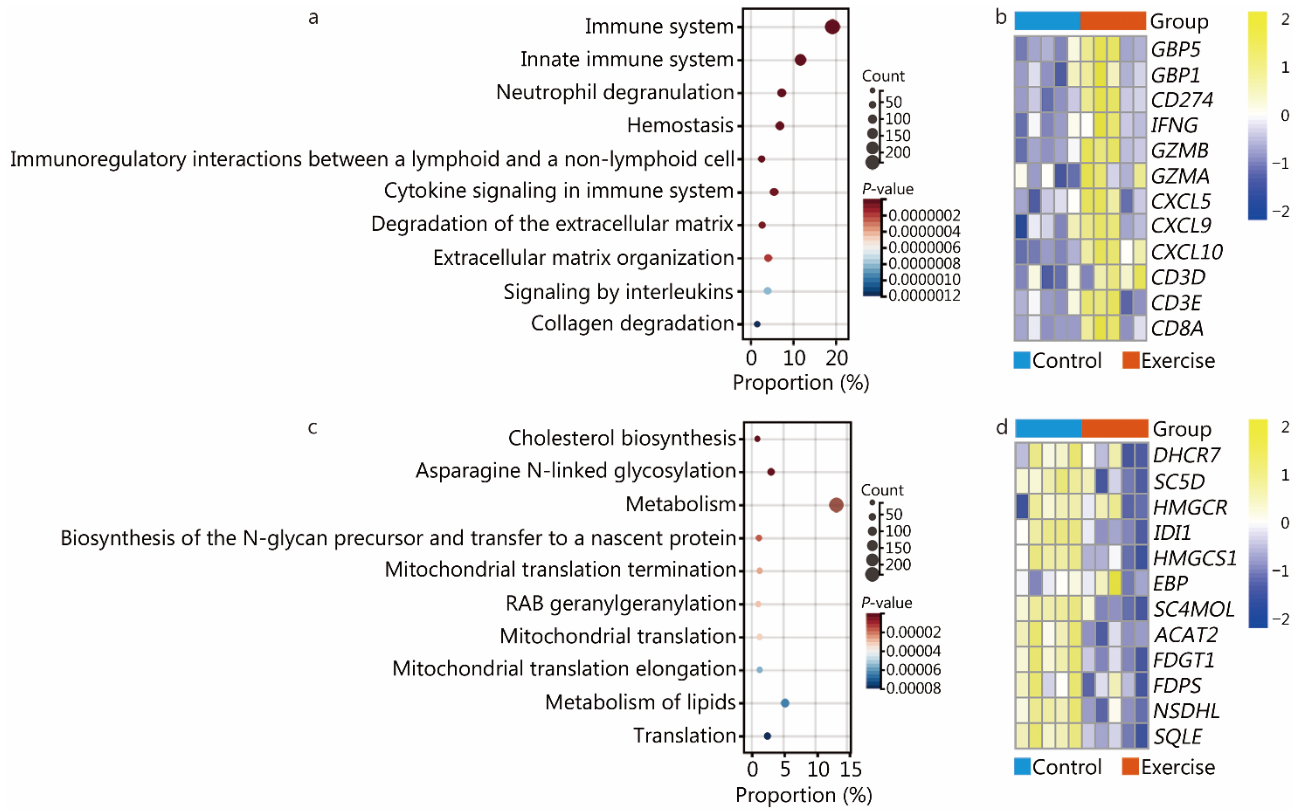

Fig. S2 Reactome analysis of DEGs in the GSE62628 dataset. **a** Reactome analysis to identify pathways that the upregulated genes were enriched in. **b** Expression levels of genes in the "immune system" module (*n* = 5 per group). **c** Reactome analysis to identify pathways that the downregulated genes were enriched in. **d** Expression levels of genes in the "cholesterol biosynthesis" module (*n* = 5 per group). DEGs differentially expressed genes, RAB one protein from RAS oncogene family, GBP guanylate binding protein, GZMA granzyme A, GZMB granzyme B, HMGCR 3-hydroxy-3-methylglutaryl-coenzyme A reductase, SQLE squalene epoxidase

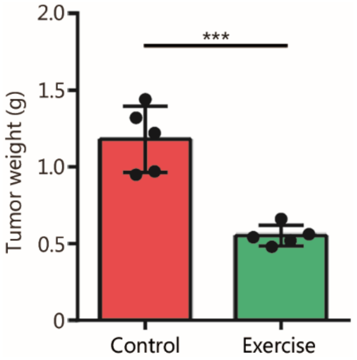

Fig. S3 Weight of the harvested tumors in tumor-bearing mice with or without exercise (n = 5 per group). $^{***}P <$ 0.001

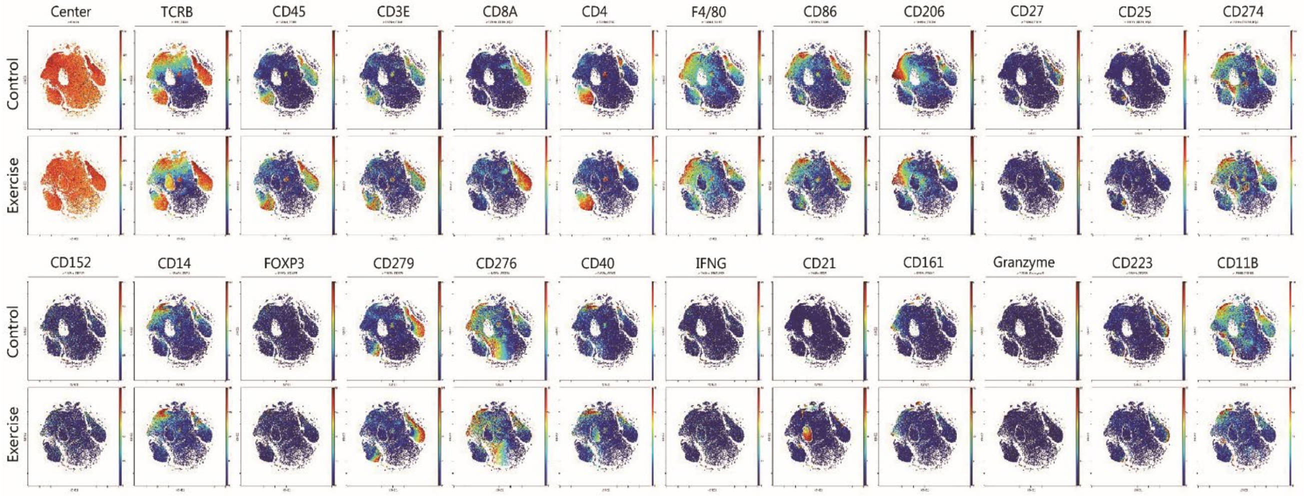

Fig. S4 t-SNE plots of normalized marker expression for classifying tumor cells from mice in the control and exercise groups. t-SNE t-distributed Stochastic Neighbor Embedding, TCRB T cell receptor beta, FOXP3 forkhead box P3, IFNG interferon gamma

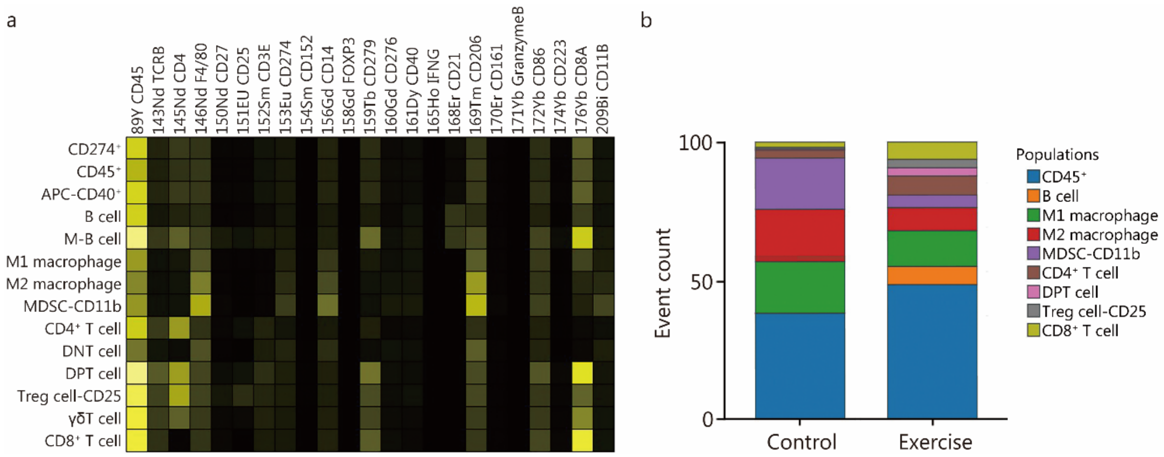

Fig. S5 Marker expression and cell proportion of different cell subpopulations. **a** Heatmap depicting the normalized expression of cell markers for 14 cell clusters. **b** Percentage of nine main cell clusters in each group. DNT double negative T cells, DPT double positive T cells, MDSC myeloid-derived suppressor cell, M-B cell memory B cell, APC antigen-presenting cells, TCRB T cell receptor beta, FOXP3 forkhead box P3, IFNG interferon gamma

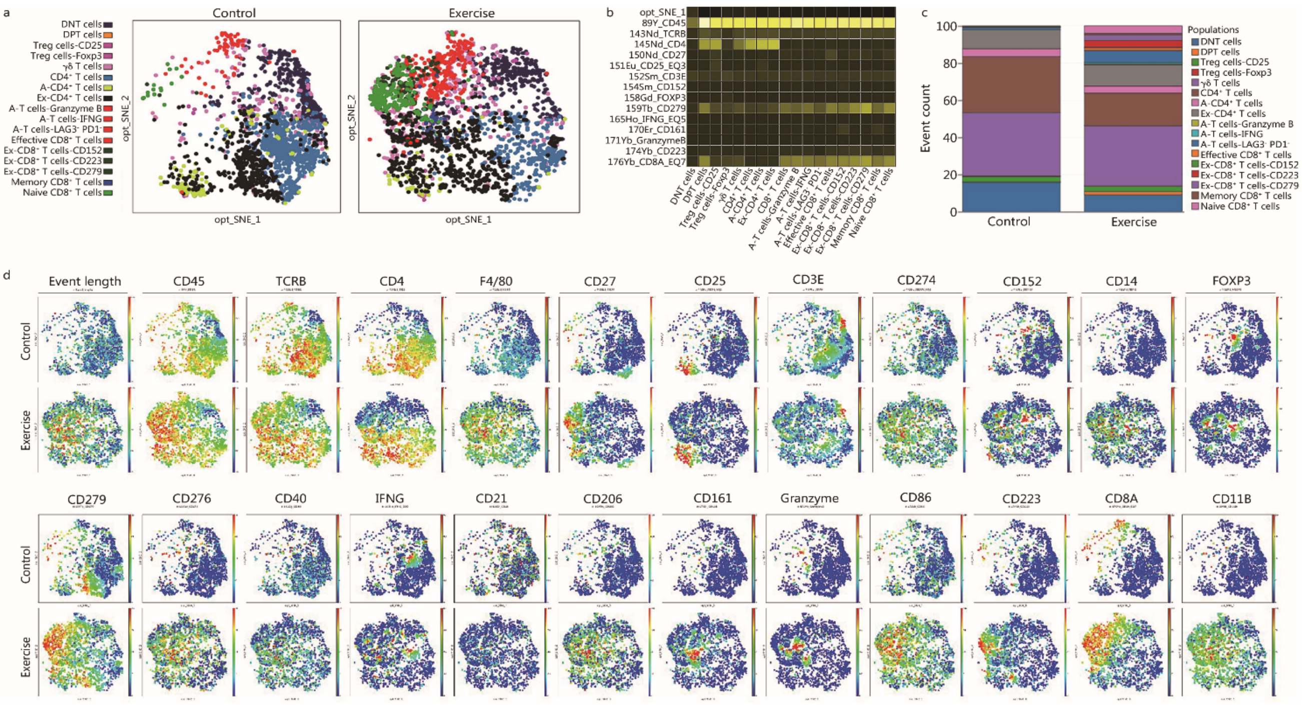

Fig. S6 Changes in the T cell subset proportions in mice tumors from the control and exercise groups. **a** Opt-SNE plots of 10,000 T cells per group, colored using PhenoGraphcluster. **b** Heatmap depicting the normalized expression of cell markers for 18 cell clusters. **c** Percentage of 17 cell clusters in each group. **d** Opt-SNE plots of normalized marker expression for classifying T cells from each group. opt-SNE optimized t-distributed Stochastic Neighbor Embedding, DNT double negative T cells, DPT double positive T cells, A-CD4/CD8^{+} T cells active CD4/CD8^{+} T cells, EX-CD4/CD8^{+} T cells exhausted CD4/CD8^{+} T cells, TCRB T cell receptor beta, FOXP3 forkhead box P3, IFNG interferon gamma

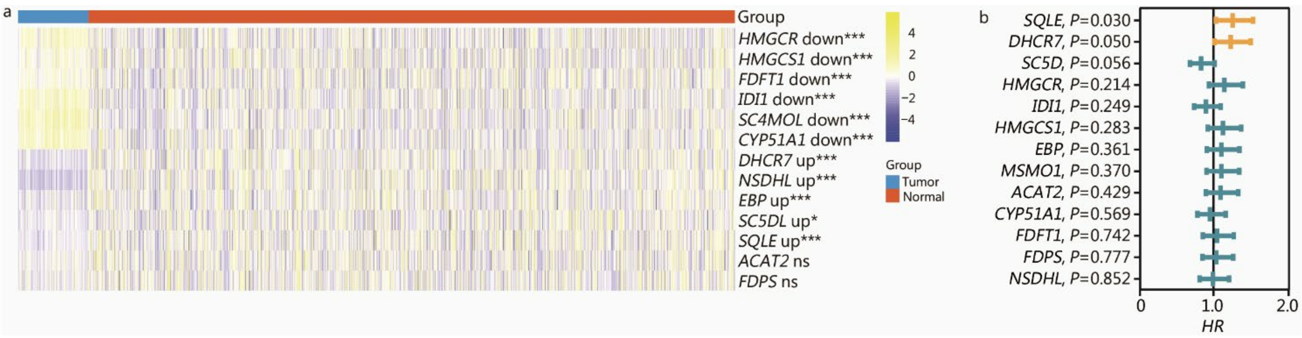

Fig. S7 Expression and prognostic value of genes related to cholesterol synthesis in NSCLC tissues. **a** Transcriptional expression levels of genes related to cholesterol synthesis in tumor and para-tumor tissues. $^{*}P$ < 0.05, $^{***}P$ < 0.001, ns non-significant. **b** Forest plot showing the prognostic values of genes related to cholesterol synthesis in NSCLC. NSCLC non-small cell lung cancer, SQLE squalene epoxidase, HMGCR 3-hydroxy-3-methylglutaryl-coenzyme A reductase

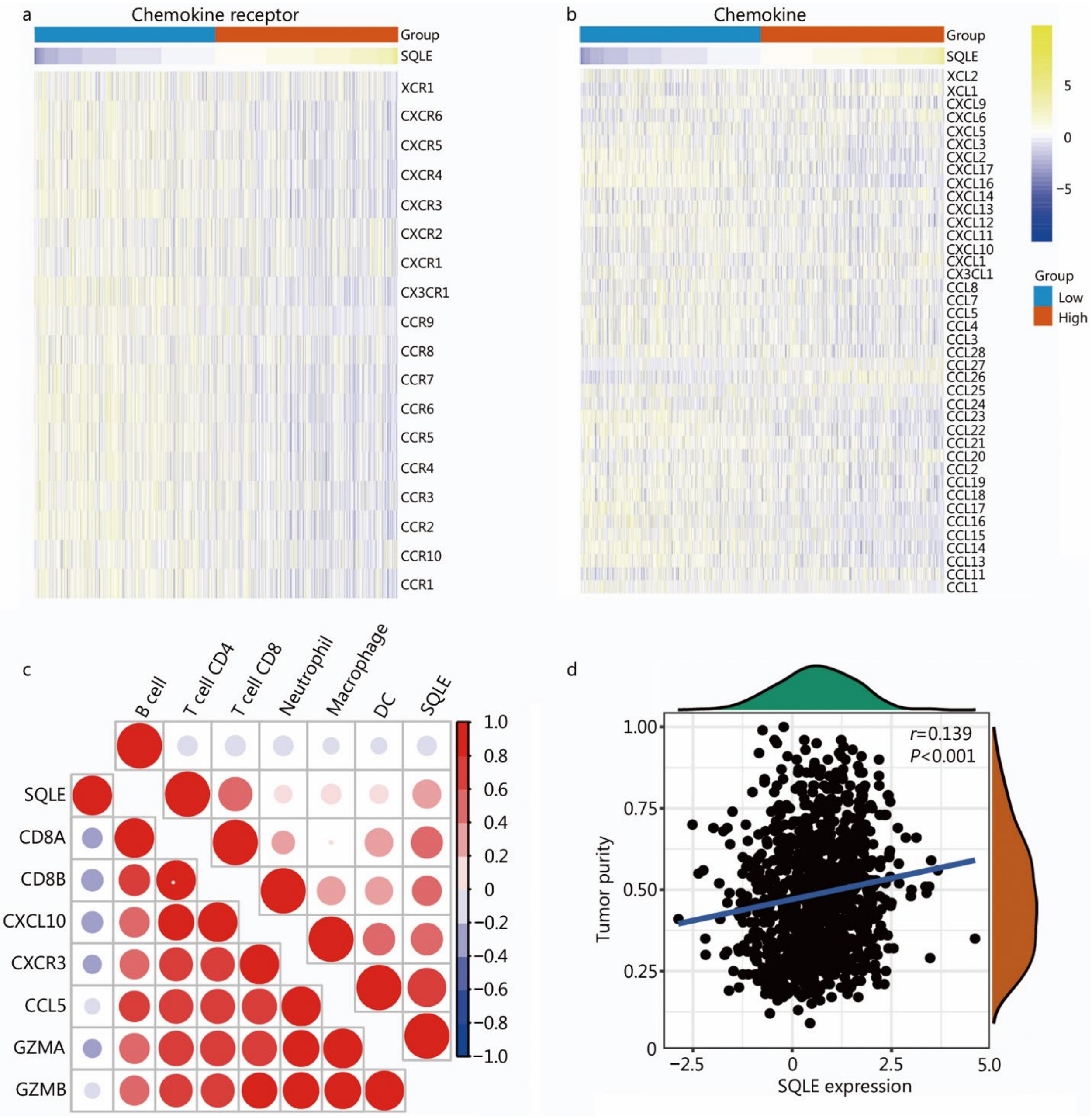

Fig. S8 Correlation between SQLE expression and TIME features. **a** and **b** Expression levels of chemokines and receptors in NSCLC in the high- and low-SQLE groups. **c** Correlation between SQLE expression and immune cell markers as well as immune cell levels estimated by the TIMER algorithm in NSCLC. **d** Correlation between SQLE expression and tumor purity in NSCLC. SQLE squalene epoxidase, TIME tumor immune microenvironment, NSCLC non-small cell lung cancer, TIMER tumor immune estimation resource, DC dendritic cells, GZMA granzyme A, GZMB granzyme B, CXCL C-X-C motif chemokine ligand, CXCR C-X-C motif chemokine receptor, CCL C-C motif chemokine ligand, CCR C-C motif chemokine receptor, XCR X-C motif chemokine receptor, XCL X-C motif chemokine ligand

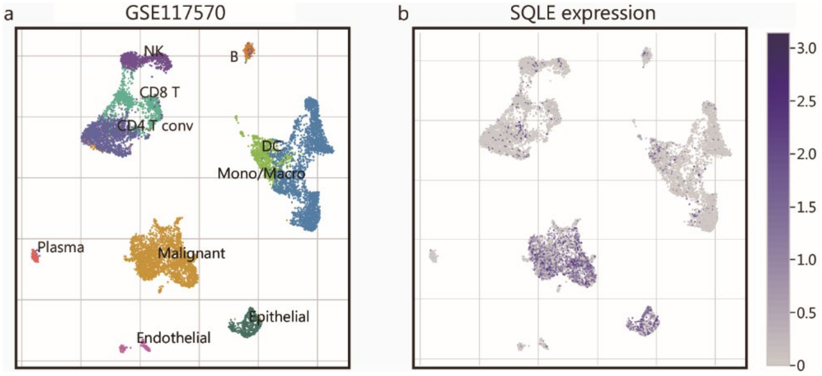

Fig. S9 Expression levels of SQLE in different cell types in NSCLC tissues in the GSE117570 dataset. **a** Distribution of cell types. **b** Expression levels of SQLE in different cell types. SQLE squalene epoxidase, NSCLC non-small cell lung cancer, Mono/Macro monocytes/macrophages, DC dendritic cells, NK natural killer cells, CD4 T conv conventional $CD4^+$ T cells

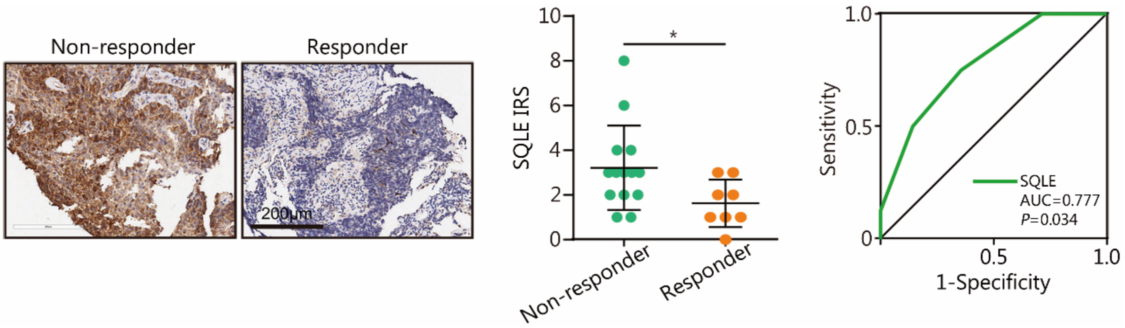

Fig. S10 Representative images depicting SQLE expression in patients with different immunotherapy responses and semi-quantitative analysis of the predictive value of SQLE (n = 14 in the non-responder group, n = 8 in the responder group). $^*P < 0.05$. SQLE squalene epoxidase, IRS immunoreactivity score

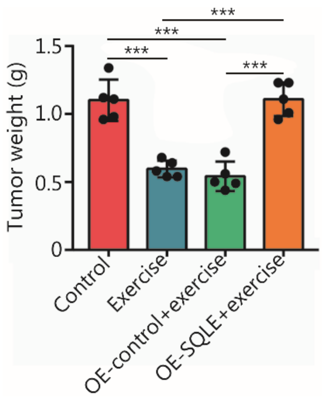

Fig. S11 Weight of the harvested tumors from tumor-bearing mice (n = 5 per group). $^{***}P < 0.001$. OE overexpression, SQLE squalene epoxidase

Table S1 A list of the antibodies and reagents used in this research

Reagent or resource	Source	Identifier
TCGA dataset	Xena	LUNG cohort
GSE135222	GEO	GSE135222
GSE126044	GEO	GSE126044
SQLE antibody	ProteinTech	12544-1-AP
HMGCR antibody	ProteinTech	13533-1-AP
CD8 antibody	GeneTech	GT2280
CD8 antibody	Abcam	ab217344
CD86 antibody	Abcam	ab220188
F4/80 antibody	Abcam	ab6640
Goat anti-rabbit IgG H&L (Alexa Fluor 488)	Abcam	ab150077
Goat anti-rat IgG H&L (Alexa Fluor 594)	Abcam	ab150160
Goat anti-mouse IgG H&L (Alexa Fluor 488)	Abcam	ab150113
Goat anti-rabbit IgG H&L (Alexa Fluor 594)	Abcam	ab150080
SQLE overexpression lentivirus	Genechem	N/A
The tissue microarray	Outdo BioTech	HLugA150CS01
The NSCLC samples	In-house cohort	N/A
DAB	Proteintech	PR30010
Hematoxylin staining solution	Beyotime	C0107
Fluorescence microscope	Echo	Revolve
Mouse lung cancer Lewis cell line	KeyGEN	KG070
Lentiviral transfection experiments	GenePharma Co., Ltd.	N/A
C57BL/6 mice	SLAC Co., Ltd.	N/A
Voluntary running wheels	Yuyan instruments	1085
DAPI	Invitrogen	D1306
Palladium ions	Fluidigm	N/A
Isothiocyanobenzyl-EDTA	Lumiprobe	M030
Fc receptor blocking reagent	BioLegend	422302
Helios mass cytometer	Fluidigm	Helios CyTOF
CD4	Fluidigm	3145002C
CD45	Fluidigm	3089005C
CD3e	Fluidigm	3152004C
CD86	Fluidigm	3172016C
F4/80	Fluidigm	3146008C

FoxP3	Fluidigm	3158003C
CD11b (Mac-1)	Fluidigm	3209003C
Ly-6G/C (Gr-1)	Fluidigm	3141005C
CD206 (MMR)	Fluidigm	3169021C
CD27	Fluidigm	3150017C
CD25 (IL-2R)	Fluidigm	3151007C
CD279 (PD-1)	Fluidigm	3159006C
CD274 (PD-L1)	Fluidigm	3153016C
CD161 (NK1.1)	Fluidigm	3170002C
CD152 (CTLA-4)	Fluidigm	3154008C
IFNg	Fluidigm	3165003C
CD223 (LAG-3)	Fluidigm	3174019C
CD14	Fluidigm	3156009C
CD21	Fluidigm	3168010C
TCRb	Fluidigm	3143010C
CD40	Fluidigm	3161020C
Granzyme B	Fluidigm	3171002C
FITC	Fluidigm	3160011C
APC	Fluidigm	3176007C
Cisplatin	Fluidigm	201064
Intercalator-Ir	Fluidigm	201192A
Maxpar cell staining buffer (500 ml)	Fluidigm	201068
Maxpar fix and perm buffer (100 ml)	Fluidigm	201067
Maxpar water (500 ml)	Fluidigm	201069
Maxpar cell acquisition solution plus for CyTOF XT-1L	Fluidigm	201244
Maxpar nuclear antigen staining buffer set	Fluidigm	201063

CyTOF cytometry by time-of-flight, *DAB* diaminobenzidine, *DAPI* 4',6-diamidino-2-phenylindole dihydrochloride, *HMGCR* 3-hydroxy-3-methylglutaryl-coenzyme A reductase, *NSCLC* non-small cell lung cancer, *SQLE* squalene epoxidase, *TCGA* The Cancer Genome Atlas, *FITC* fluorescein isothiocyanate isomer I, *APC* allophycocyanin, *IFNg* interferon gamma, *CTLA-4* cytotoxic T-lymphocyte associated protein 4, *MMR* macrophage mannose receptor, *PD-1* programmed cell death protein 1, *LAG-3* lymphocyte activation gene-3
